# NK cells of the oldest seniors represent constant and resistant to stimulation high expression of cellular protective proteins SIRT1 and HSP70

**DOI:** 10.1186/s12979-018-0115-x

**Published:** 2018-03-06

**Authors:** Lucyna Kaszubowska, Jerzy Foerster, Jan Jacek Kaczor, Daria Schetz, Tomasz Jerzy Ślebioda, Zbigniew Kmieć

**Affiliations:** 1 Department of Histology, Medical University of Gdańsk, Dębinki 1, 80-211 Gdańsk, Poland; 2 Department of Social and Clinical Gerontology, Medical University of Gdańsk, Dębinki 1, 80-211 Gdańsk, Poland; 3 Department of Bioenergetics and Physiology of Exercise, Medical University of Gdańsk, Dębinki 1, 80-211 Gdańsk, Poland; 4 Department of Pharmacology, Medical University of Gdańsk, Dębowa 23, 80-204 Gdańsk, Poland

**Keywords:** NK cells, Ageing, Adaptive stress response, SIRT1, HSP70, SOD2, Isoprostanes, Carbonyl groups, Oxidative stress, Innate immunity

## Abstract

**Background:**

Natural killer cells (NK cells) are cytotoxic lymphocytes of innate immunity that reveal some immunoregulatory properties, however, their role in the process of ageing is not completely understood. The study aimed to analyze the expression of proteins involved in cellular stress response: sirtuin 1 (SIRT1), heat shock protein 70 (HSP70) and manganese superoxide dismutase (SOD2) in human NK cells with reference to the process of ageing. Non-stimulated and stimulated with IL-2, LPS or PMA with ionomycin cells originated from peripheral blood samples of: seniors aged over 85 (‘the oldest’; *n* = 25; 88.5 ± 0.5 years, mean ± SEM), seniors aged under 85 (‘the old’; *n* = 30; 75.6 ± 0.9 years) and the young (*n* = 31; 20.9 ± 0.3 years). The relationships between the levels of expression of cellular protective proteins in the studied population were also analyzed. The concentrations of carbonyl groups and 8-isoprostanes, markers of oxidative stress, in both stimulated and non-stimulated cultured NK cells were measured to assess the level of the oxidative stress in the cells.

**Results:**

The oldest seniors varied from the other age groups by significantly higher expression of SIRT1 and HSP70 both in non-stimulated and stimulated NK cells. These cells also appeared to be resistant to further stimulations with IL-2, LPS or PMA with ionomycin. Highly positive correlations between SIRT1 and intracellular HSP70 in both stimulated and non-stimulated NK cells were observed. SOD2 presented low expression in non-stimulated cells, whereas its sensitivity to stimulation increased with age of donors. High positive correlations between SOD2 and surface HSP70 were observed. We found that the markers of oxidative stress in NK cells did not change with ageing.

**Conclusions:**

The oldest seniors revealed well developed adaptive stress response in NK cells with increased, constant levels of SIRT1 and intracellular HSP70. They presented also very high positive correlations between expression of these cellular protective proteins both in stimulated and non-stimulated cells. These phenomena may contribute to the long lifespan of this group of elderly. Interestingly, in NK cells SOD2 revealed a distinct role in cellular stress response since it showed sensitivity to stimulation increasing with age of participants. These observations provide novel data concerning the role of NK cells in the process of ageing.

## Background

Natural killer cells (NK cells) are cytotoxic lymphocytes of innate immune system. They are cytotoxic ILCs1 (innate lymphoid cells type 1) crucial for immune response against viral infection and tumor cells [1, 2] but demonstrate also some immunoregulatory activities by secretion of cytokines and chemokines [3, 4]. They may adjust to the alterations of the cellular environment and develop a type of antigen-specific immunological memory exposing characteristics of both innate and adaptive immunity [5, 6]. After activation by cytokines such as IL-2, IL-12, IL-15 and IL-18 in different combinations or target cell challenge they secrete a range of cytokines, e.g. TNF, IFN-γ, IL-5, IL-10, IL-13, GM-CSF and chemokines IL-8, MIP-1α, MIP-1β and RANTES [7–9].

Interleukin 2 is one of the key NK cell cytokines required for the survival, proliferation and activation of MKK/ERK pathway, which was shown to be necessary for the activation of NK cells, IFN-γ secretion, CD25 and CD69 expression and enhanced cytotoxic function [10].

NK cells can be also activated by lipopolysaccharide (LPS), a component of the outer membrane of Gram-negative bacteria [11, 12]. LPS is recognized by Toll-like receptors 4 (TLR4) which play a crucial role in innate immunity and are expressed also on the surface of NK cells [13, 14]. TLR4 interacts by binding lipid A, a part of LPS molecule what results in activation of NF-κB pathway and expression of genes coding for proinflammatory cytokines, e.g. TNF, IL-1, IL-6, GM-CSF and chemokines, e.g. IL-8, RANTES, MIP-1α, MCP-1 [15, 16].

Phorbol 12-myristate 13-acetate (PMA) is a protein kinase C (PKC) activator used for a strong and unspecific stimulation of NK cells. Ionomycin (Ca^2+^ ionophore A23187) is a calcium ion channel opening antibiotic that mimicks the action of inositol triphosphate (IP3) and increases intracellular cytoplasmic free Ca^2+^ concentration by causing an influx of calcium ions from the extracellular space into cell cytoplasm [17]. Ca^2+^-PKC pathway is involved in the phosphorylation of STAT4 which binds to numerous sequences in the genome including promoters of proinflammatory cytokine genes such as IFN-γ and TNF [18]. PMA with ionomycin are used for short (1-6 h) cell stimulation to induce the expression of cytokines, e.g. IFN-γ [19–21] and for both short [19] and longer (up to 48 h) cell stimulations to analyze profiles of gene and protein expression [22, 23].

Ageing is associated with the progressive increase in the proinflammatory status caused by the general decrease in the capacity to cope with a variety of stress factors and impairment of regulatory mechanisms concerning cytokine secretion. In ageing increased serum level of IL-6 and TNF, elevated level of C reactive protein and decreased concentration of anti-inflammatory cytokine IL-10 have been often reported [24]. These phenomena have been described as “inflamm-aging” [25]. However, ageing is also characterized by the increase of oxidative stress due to the redox imbalance caused by discrepancy between the amount of reactive oxygen species (ROS) generation and the activity of anti-oxidative mechanisms [26]. Thus, the theory of oxidation-inflammation was proposed as the main cause of ageing and the new term “oxi-inflamm-aging” was introduced to accentuate the relation between the redox state and functional capacity of the ageing immune system [27].

Adaptive stress response of cells and organisms to an intracellular or extracellular moderate stress is referred to the term hormesis used in toxicology to reflect a dose response to drugs, toxins or some natural substances. Low doses of these substances may elicit a positive response regarding adaptation to or protection from the stress agents, whereas at higher concentrations the same substances reveal toxic effects [28, 29]. The cellular adaptive response usually contributes to the synthesis of various stress resistance proteins, such as heat shock proteins, sirtuins, heme oxygenase and thioredoxin system [29].

SIRT1 is a redox sensitive protein that protects cells against cellular senescence caused by oxidative stress [30]. It is a (NAD^+^) - dependent deacetylase that targets a variety of transcription factors, including FOXO1, 3 and 4, p53, NF-κB, PGC-1 and HSF-1. These factors control cellular stress adaptive responses which can then modulate the lifespan [31]. Expression of chaperons that protect cells against cellular stress is under control of heat shock factor-1 (HSF1), which is activated within minutes after appearing of the stress agent [32–34]. The presence of HSF-1 in the stress regulatory network indicates the central role of protein homeostasis in SIRT1-mediated cellular protection [35]. Manganese superoxide dismutase (SOD2) is a major mitochondrial enzymatic antioxidant which is under control of FOXO1, FOXO3a, FOXO4 and NF-κB transcription factors activated in cellular stress adaptive responses [36, 37].

The changes in the expression of SIRT1 in PBMCs [38] or HSP70 in granulocytes [39] or monocytes and lymphocytes [40, 41] have been described in the process of ageing. However, there are no studies regarding this phenomenon in NK cells, lymphocytes associated with healthy ageing and longevity [42, 43]. Therefore, the aim of our study was to analyze the expression of the proteins involved in cellular stress response: sirtuin 1, heat shock protein 70 and manganese superoxide dismutase in both non-stimulated and stimulated with IL-2, LPS and PMA with ionomycin human NK cells of the young, seniors under 85 and the oldest seniors aged over 85. We studied both the influence of the process of stimulation on the level of cellular protective proteins and the potential relationships between these proteins in the process of ageing. The levels of oxidative stress markers were also measured both in stimulated and control (non-stimulated) NK cells.

## Methods

### Participants

Eighty six volunteers aged between 19 and 94 years (62 women and 24 men) participated in this study. The exclusion criteria included: CRP > 5 mg/L, cancer, autoimmune disease, diabetes, infection, use of immunosuppressive drugs, glucocorticoids or non-steroid anti-inflammatory drugs (NSAID). Absence of dementia was assessed using the “Mini Mental State Examination” and only seniors with the score above 23 points were qualified to the study [44]. All senior volunteers underwent a geriatric assessment. The Katz’s index of independence in “Activities of Daily Living” (ADL) was used and only seniors with 5-6 points were enrolled to the study [45]. Senior volunteers were recruited among inhabitants of local retirement homes whereas young volunteers were students of Medical University of Gdańsk, Poland. The participants were subdivided into 3 age groups: young (range 19-24 years), old (seniors aged under 85; range 65-84 years) and the oldest (seniors at the age over 85; range 85-94 years). The characteristics of the study population are shown in Table 1. All volunteers signed informed consent and the study received approval from Ethical Committee of Medical University of Gdańsk, Poland (No 225/2010). An immunological characteristics of the study population was described earlier [46].

**Table 1:** Characteristics of the study population

Parameter	Young	Old	Oldest
Number	31	30	25
Age (yr)	20.9 ± 0.3	75.6 ± 0.9	88.5 ± 0.5
Sex (F/M)	22/9	20/10	20/5
Smoking status			
Current smoker	6	3	1
Ex-smoker	3	3	0
Nonsmoker	22	24	24
Weight (kg)	59.8 ± 1.7^a,b^	68.7 ± 2.0^a^	68.4 ± 2.3^b^
BMI (Body Mass Index)	20.4 ± 0.4^a,b^	25.6 ± 0.6^a^	26.5 ± 0.9^b^
Total cholesterol (mg/dL)	170.5 ± 5.6^a^	193.6 ± 8.3^a^	184.1 ± 9.4
HDL – cholesterol (mg/dL)	58.7 ± 2.6^a,b^	47.3 ± 2.3^a^	43.7 ± 2.2^b^
LDL – cholesterol (mg/dL)	96.1 ± 4.3^a^	123.3 ± 7.3^a^	113.7 ± 6.9
Triglyceride (mg/dL)	78.3 ± 8.2^a,b^	114.8 ± 11.2^a^	133.0 ± 14.8^b^
Glucose (mg/dL)	89.35 ± 1.33	94.4 ± 3.3	91.36 ± 3.4
Creatinine (mg/dL)	0.86 ± 0.03^b^	0.87 ± 0.04^c^	1.05 ± 0.05^b c^
Uric acid (mg/dL)	4.95 ± 0.21^b^	5.37 ± 0.28	6.22 ± 0.36^b^

All data are presented as means ± SEM. Statistically significant differences between age groups are marked with:

^a^young vs old

^b^young vs oldest

^c^old vs oldest

### Preparation of peripheral blood mononuclear cell cultures

Peripheral blood mononuclear cells (PBMCs) were isolated from venous blood samples collected in tubes with EDTA by conventional ficoll-uropoline density gradient centrifugation. PBMCs were then washed and resuspended in RPMI1640 medium supplemented with 5% FBS, penicillin (100 U/ml) – streptomycin (100 μg/ml) and 2-mercaptoethanol (5 × 10^− 5^ M) (all purchased from Sigma - Aldrich, Saint Louis, MO, USA). Cells (5 × 10^5^ / 0.5 ml) were cultured for 48 h in the absence (control) or presence of IL-2 (100 U/ml) (BD Biosciences, San Jose, CA, USA), LPS (1 μg/ml) or PMA (50 ng/ml) and ionomycin (500 ng/ml, all purchased from Sigma-Aldrich). PBMCs treated in this way were studied for the expression of SIRT1, SOD2 and HSP70 (surface and intracellular). The intracellular expression of TNF and IFN-γ, was studied in PBMCs (5 × 10^5^ / 0.5 ml) cultured in the absence (control) or presence of IL-2 (100 U/ml) (BD Biosciences, San Jose, CA, USA), LPS (1 μg/ml) (Sigma-Aldrich, Saint Louis, MO, USA) or PMA (50 ng/ml) and ionomycin (500 ng/ml) (Sigma - Aldrich, Saint Louis, MO, USA) for 5 h. Simultaneously, Golgi Stop reagent (0.5 μl / well in 0.5 ml of medium, BD Biosciences, San Jose, CA, USA) was added to PBMC cultures (5 × 10^5^ / 0.5 ml) to stop extracellular export of cytokines. Then PBMCs were collected and washed with 1 ml of BD Staining Buffer.

### Staining of surface and intracellular antigens for flow cytometry

PBMCs (2.5 × 10^5^ cells) were aliquoted into flow cytometry tubes and CD3-FITC-conjugated (0.125 μg/ml; clone UCHT1) (BD Biosciences, San Jose, CA, USA) or CD3-PE-Cy7-conjugated (0.125 μg/ml; clone SK7) (BD Biosciences, San Jose, CA, USA), CD56-APC-conjugated (0.6 μg/ml; clone NCAM16.2) (BD Biosciences, San Jose, CA, USA) and Hsp70-PE-conjugated (1 μg/ml; clone N27F34) (Abcam, Cambridge, England) monoclonal antibodies were added for cell surface antigen staining. After 30 min of incubation in the dark at room temperature 2 ml of BD FACS Lysing Solution was added and samples were incubated for subsequent 10 min in the same conditions. Then cells were washed twice with 1 ml of BD Staining Buffer (PBS without Ca^2+^ and Mg^2+^, 1% FBS, 0.09% sodium azide) and resuspended in 0.25 ml of Fixation/Permeabilization Solution for 20 min at 4 °C following manufacturer’s protocol (BD Cytofix/Cytoperm Fixation/Permeabilization Kit). Cells were washed twice with 1 ml of BD Perm/Wash buffer and relevant volumes of MnSOD-FITC-conjugated (1 μg/ml; clone MnS-1) (eBioscience, San Diego, CA, USA), Hsp70-PE-conjugated (1 μg/ml; clone N27F34) (Abcam, Cambridge, England), SIRT1-Alexa Fluor 488 – conjugated (1 μg/ml; clone 19A7AB4) (Abcam, Cambridge, England), TNF-PE-Cy7- conjugated (0.125 μg/ml; clone MAb11) (BD Biosciences, San Jose, CA, USA) or IFN-γ-PE-conjugated (0.125 μg/ml; clone 4S.B3) (BD Biosciences, San Jose, CA, USA) monoclonal antibodies were added for staining of intracellular antigens following the manufacturer’s instructions. After 30 min of incubation in the dark at room temperature cells were washed twice with 1 ml of BD Perm/Wash buffer and resuspeded in Staining Buffer prior to flow cytometric analysis. Samples were run on a BD FACSCalibur flow cytometer equipped with argon-ion laser (488 nm) and data were evaluated with BD CellQuest Pro software (BD Biosciences, San Jose, CA, USA) after collecting 10,000 gated events (lymphocytes). Peripheral blood lymphocytes were gated using forward (FSC) and side scatter (SSC) parameters. NK cells were identified in the CD3-negative region based on the expression of CD56 surface marker and defined as CD3-CD56+ cells. NK cell subset, gated on CD3-CD56+ cells, was further analyzed for the frequency of cells expressing the particular cellular protective protein (SIRT1, HSP70, SOD2) or cytokine (TNF and IFN-γ). Relevant isotype controls for both surface and intracellular staining were also used.

### Separation of NK cells for measurement of protein carbonyl groups and 8-isoprostane concentrations in cell lysates

NK cells (CD3^−^CD56^+^) were isolated from PBMCs by negative selection with the use of Human NK Cell Enrichment Kit and EasySep Magnet (Stemcell Technologies, Vancouver, Canada). PBMCs were incubated with EasySep Human NK Cell Enrichment Cocktail (a suspension of monoclonal antibodies bound in bispecific Tetrameric Antibody Complexes (TAC) directed against cell surface antigens on human blood cells: CD3, CD4, CD14, CD19, CD20, CD36, CD66b, CD123, HLA-DR, glycophorin A and dextran for 10 min, then vortexed for 30 s and incubated with EasySep D Magnetic Particles (a suspension of magnetic dextran iron particles) for subsequent 5 min. Then cells were resuspended in 2.5 ml of recommended medium (PBS with 2% FBS and 1 mM EDTA, Ca^2+^ and Mg^2+^ free), mixed gently and placed into the magnet. After 2.5 min the desired fraction was poured off into a new tube. Aliquots of the cell fractions were stained with relevant volumes of CD56-PE- and CD3-PerCP-conjugated monoclonal antibodies (BD Biosciences, San Jose, CA, USA). After 30 min of incubation in the dark at room temperature cells were washed with 2 ml of BD CellWASH solution and finally 0.5 ml of BD CellFIX solution was added. Samples were stored at 4 °C up to 24 h until analyzed by flow cytometry to check the purity of the enriched NK cell fractions and all showed almost 95% purity.

Then, NK cell extracts were prepared with the use of Mammalian Cell & Tissue Extraction Kit (BioVision Research Products, Mountain View, CA, USA) following the manufacturer’s protocol. The total protein concentration of samples was estimated with Bradford assay (Sigma - Aldrich, Saint Louis, MO, USA). Cell lysates were stored at − 70 °C for further analysis. The content of protein carbonyl groups in NK cell extracts was determined with the BioCell PC Test Kit, an enzyme-linked immunosorbent assay (BioCell, Auckland, New Zealand). Samples were provided for ELISA procedure following the manufacturer’s instructions. Absorbances were measured at 450 nm with Bio-Rad plate reader. A standard curve reflecting absorbances of the increasing concentrations of protein carbonyls in the supplied oxidized protein standards was made and used to determine the concentration of carbonyl groups in the analyzed samples. Data in the study are expressed as nanomoles of carbonyl groups per mg of cellular extract protein (nmol/mg).

The concentration of 8-isoprostanes in NK cell extracts was determined with the 8-Isoprostane ELISA Kit (Cayman Chemical, Ann Arbor, MI, USA). Samples were provided for ELISA procedure following manufacturer’s instructions. Cell lysates were supplied in the presence of 0.005% BHT. Absorbances were measured at 405 nm with Bio-Rad plate reader. A standard curve was made with the use of 8-Isoprostane ELISA Standard provided with the kit and the concentrations of 8-isoprostanes in the analyzed samples were evaluated. Data in the study present the total 8-isoprostane content in NK cell lysates expressed as pg/ml.

### Statistics

All data are expressed as means ± SEM. Normality of data distribution was analyzed by Shapiro-Wilk test. ANOVA test for normal distribution and Kruskal-Wallis test for non-parametric distribution were used to compare experimental data. The multiple comparisons were performed with Tukey’s post-hoc test for normal distribution and Dunn’s post-hoc test for non –parametric distribution. Paired Student’s t-test for normal distribution and Wilcoxon signed-rank test for non-parametric distribution were used to compare two related samples. Student’s t test for normal distribution and Mann-Whitney U test for non-parametric distribution were used to compare two independent samples. The Spearman correlation coefficient (R) was applied to present the strength of the relationship between variables (Statistica, version 12; Statsoft, Tulsa, OK, USA). Differences and correlations with *p* < 0.05 were considered as statistically significant.

## Results

### Expression of SIRT1 and HSP70^intracellular^ in non- stimulated and stimulated NK cells of the seniors and the young

**Fig. 1:**
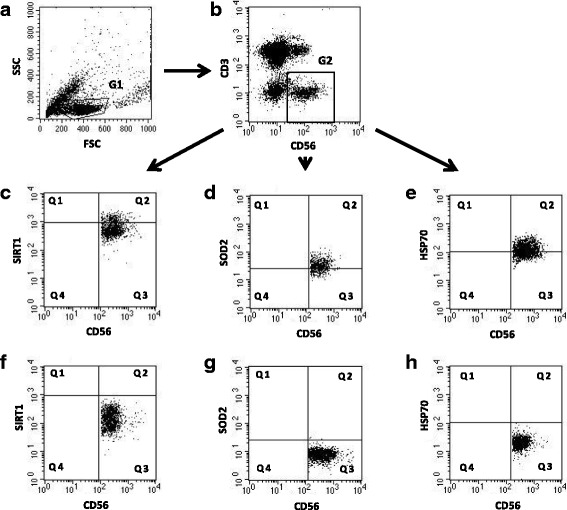
Gating strategy for flow cytometric analyses of NK cells. **a** Forward scatter (FSC) and side scatter (SSC) characteristics of PBMC population with gated lymphocytes (G1). **b** NK cell gating – NK cells were defined as CD3 negative and CD56 positive population (G2). **c** NK cells expressing SIRT1 were identified in the upper right quadrant (Q2). **d** NK cells expressing SOD2 were identified in the upper right quadrant (Q2). **e** NK cells expressing intracellular HSP70 were identified in the upper right quadrant (Q2). **f** Isotype control for SIRT1 positive cells (Q3). **g** Isotype control for SOD2 positive cells (Q3). **h** Isotype control for HSP70 positive cells (Q3)

The gating strategy performed for flow cytometric analysis of NK cells is demonstrated in Fig. 1. Flow cytometry data were analyzed and presented in two ways, i.e. as the percentage of NK cells showing the expression of the studied protein (% of positive cells) and mean fluorescence intensity (MFI) measured in the samples.

**Fig. 2:**
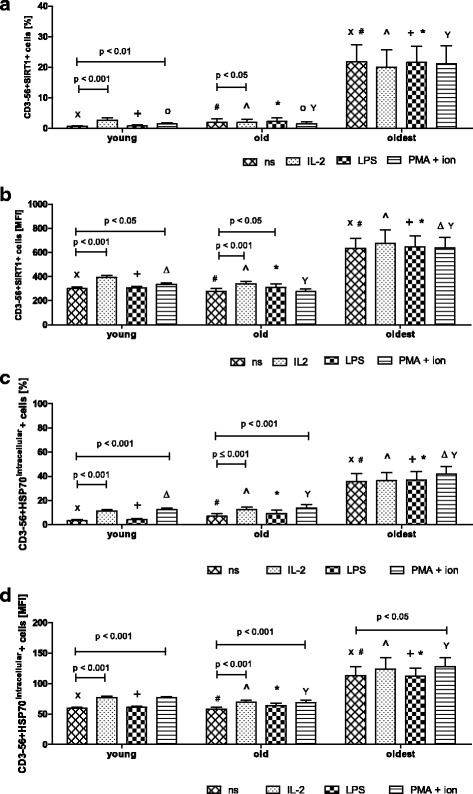
Expression of SIRT1 and intracellular HSP70 in non-stimulated and stimulated NK cells of the young, the old aged under 85 and the oldest seniors (aged over 85). Data are presented as means ± SEM and show expression of the studied proteins in NK cells demonstrated as percentages of cells with the expression of a particular protein (%) or mean fluorescence intensity (MFI). The same symbols over bars (^X^, ^+^, ^o^, ^#^, *, ^, ^Δ^, ^Y^) denote statistically significant differences between similarly treated NK cells of different age groups (i.e. young vs old; young vs oldest or old vs oldest). Horizontal lines above paired bars denote statistically significant differences between treated and untreated NK cells of the same age group (i.e. non-stimulated vs stimulated with IL-2, LPS or PMA with ionomycin). **a** Expression of SIRT1 (%): ^X^, ^#^, ^, ^+^, *, ^Y^*p* < 0.001; ^o^*p* ≤ 0.01. **b** Expression of SIRT1 (MFI): ^#^, ^+^, *, ^Y^*p* < 0.001; ^, ^X^*p* < 0.01; ^Δ^*p* < 0.05. **c** Expression of HSP70^intracellular^ (%): ^X^, ^+^, ^Y^*p* < 0.001; ^#^, *, ^Δ^*p* < 0.01; ^ *p* < 0.05. **d** Expression of HSP70^intracellular^ (MFI): ^#^, ^+^, ^Y^, * *p* ≤ 0.001; ^X^, ^ *p* ≤ 0.01

In the group of the oldest seniors 20% of NK cells independently on the presence or absence of stimulation revealed increased intracellular expression of SIRT1 (range 19.98 ± 5.77% – 21.82 ± 5.56%). It was significantly lower in the group of seniors under 85 (range 1.37 ± 0.77% – 2.22 ± 1.33%) and in the young (range 0.64 ± 0.24% – 2.64 ± 0.8%) in all studied conditions (Fig. 2a). In contrast to NK cells of the younger subjects, NK cells of the oldest seniors were not sensitive to any type of the applied method of stimulation (Fig. 2a, b). In the young, NK cells were sensitive to stimulation with IL-2 and PMA with ionomycin (Fig. 2a, b). In the elderly under 85, NK lymphocytes were sensitive to IL-2 (Fig. 2a) or IL-2 and LPS (Fig. 2b), although in general the incubation with LPS revealed rather a limited influence on the expression level of SIRT1 in the cells (Fig. 2a, b).

It was also noteworthy that nearly 40% of NK cells of the oldest seniors showed increased expression of HSP70^intracellular^ in all studied samples (range 35.6 ± 6.8% - 41.7 ± 6.28%) on the contrary to NK cells of the old (range 6.87 ± 2.37 – 13.61 ± 2.76%) and the young (range 3.28 ± 0.77% - 12.32 ± 1.34%) (Fig. 2c). Out of the studied stimulating agents only PMA with ionomycin increased HSP70^intracellular^ expression in NK cells of the oldest subjects (Fig. 2d). Similarly to SIRT1, the expression of HSP70^intracellular^ in NK cells of the young and the old was sensitive to stimulation with IL-2 and PMA with ionomycin, whereas the effect of LPS was very limited (Fig. 2c, d).

### Expression of SOD2 and HSP70^surface^ in non-stimulated and stimulated NK cells of the seniors and the young

**Fig. 3:**
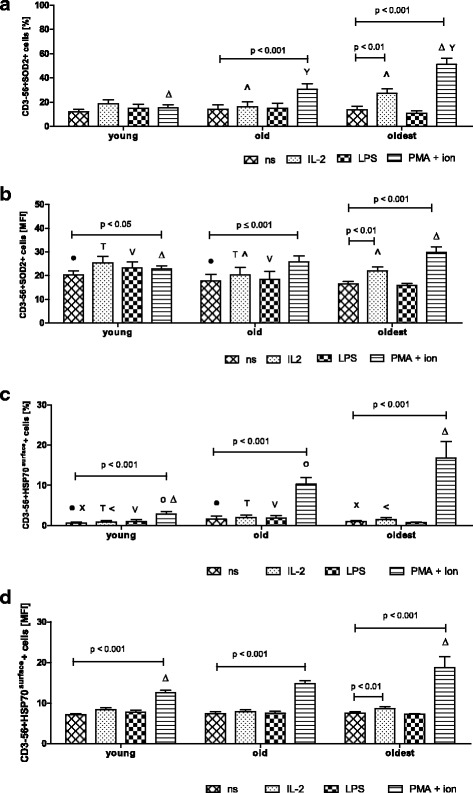
Expression of SOD2 and surface HSP70 in non-stimulated and stimulated NK cells of the young, the old aged under 85 and the oldest seniors (aged over 85). Data are presented as means ± SEM and concern expression of the studied proteins in NK cells demonstrated as percentages of cells with expression of a particular protein (%) or mean fluorescence intensity (MFI). The same symbols over bars ^X^, ^<^, ^●^, ^T^, ^o^, ^V^, ^, ^Δ^, ^Y^ denote statistically significant differences between similarly treated NK cells of different age groups (i.e. young vs old; young vs oldest or old vs oldest). Horizontal lines above paired bars denote statistically significant differences between treated and untreated NK cells of the same age group (i.e. non-stimulated vs stimulated with IL-2, LPS or PMA with ionomycin). **a** Expression of SOD2 (%): ^Δ^*p* < 0.001; ^, ^Y^*p* < 0.01. **b** Expression of SOD2 (MFI): ^T^, ^, ^V^*p* < 0.01; ^Δ^, ^●^ *p* < 0.05. **c** Expression of HSP70^surface^ (%): ^X^, ^o^, ^Δ^*p* < 0.001; ^T^, ^<^, ^V^, ^●^ *p* < 0.01. **d** Expression of HSP70^surface^ (MFI): ^Δ^*p* ≤ 0.001

The intracellular expression of the other cytoprotective protein SOD2 revealed a different pattern compared to the expression of SIRT1 and HSP70^intracellular^. The NK cells of the oldest seniors appeared to be the most sensitive to the stimulation. We observed a significant increase in SOD2 expression after stimulation with IL-2 (twofold) and PMA with ionomycin (nearly fourfold) compared to non-stimulated cells (13.8 ± 2.69%) (Fig. 3a). In the elderly below 85 years NK cells were sensitive to the stimulation with PMA with ionomycin and revealed twofold increase in SOD2 expression compared to non-stimulated cells (14.0 ± 3.54%). In NK cells of the young, however, we did not observe any alterations in SOD2 expression after stimulation (Fig. 3a). These results were also confirmed by the measurements of the mean fluorescence intensity (MFI) (Fig. 3b). The only differences between these two analyses concerned the young subjects as NK cells of the young stimulated with PMA and ionomycin showed a significant increase in the expression level of SOD2 in the analysis of MFI (Fig. 3b).

The extracellular HSP70 (HSP70^surface^) was expressed in NK cells of all age groups after stimulation at a very low level, comparable to resting NK cells, except for NK cells stimulated with PMA and ionomycin (Fig. 3c). The combination of these two agents increased the expression of HSP70^surface^ in all studied age groups: in the young fourfold, in the old sixfold and in the oldest nearly sixteen-fold compared to the level in non-stimulated cells; i.e. 0.66 ± 0.27% in the young, 1.65 ± 0.76% in the old and 1.07 ± 0.21% in the oldest (Fig. 3c). Similar results were obtained after the analysis of MFI parameter specific for the studied groups. This method revealed additionally the susceptibility of NK cells of the oldest seniors to the stimulation with IL-2 (Fig. 3d). LPS did not influence significantly the expression level of SOD2 and HSP70^surface^ (Fig. 3a-d).

### Expression of TNF and IFN-γ in non-stimulated and stimulated NK cells of the seniors and the young

**Fig. 4:**
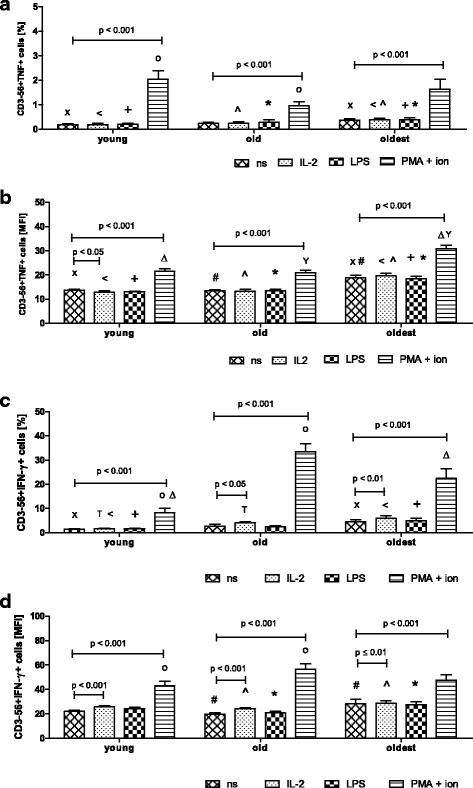
Expression of TNF and IFN-γ in non-stimulated and stimulated NK cells of the young, the old aged under 85 and the oldest seniors (aged over 85). Data are presented as means ± SEM and concern expression of the studied proteins in NK cells demonstrated as percentages of cells with expression of a particular protein (%) or mean fluorescence intensity (MFI). The same symbols over bars ^X^, ^<^, ^T^, ^o^, ^, ^Δ^, ^+^, *, ^#^, ^Y^ denote statistically significant differences between similarly treated NK cells of different age groups (i.e. young vs old; young vs oldest or old vs oldest). Horizontal lines above paired bars denote statistically significant differences between treated and untreated NK cells of the same age group (i.e. non-stimulated vs stimulated with IL-2, LPS or PMA with ionomycin): **a** Expression of TNF (%): ^, ^<^ p ≤ 0.001; *, ^X^*p* < 0.01; ^o^,^+^*p* < 0.05. **b** Expression of TNF (MFI): ^X^, ^#^, ^<^, ^, ^+^, *, ^Δ^, ^Y^*p* < 0.001. **c** Expression of IFN-γ (%): ^<^, ^o^ *p* < 0.001; ^Δ^, ^X^, ^T^*p* < 0.01;^+^*p* < 0.05 **d** Expression of IFN-γ (MFI): ^#^, *, ^o^*p* ≤ 0.01; ^ *p* < 0.05

The expression of TNF in NK cells of all studied age groups increased significantly after stimulation with PMA and ionomycin; i.e. in the young nearly elevenfold and in the old and the oldest fourfold in relation to the level of non-stimulated cells (respectively 0.19 ± 0.04%, 0.24 ± 0.05% and 0.37 ± 0.06%,) (Fig. 4a). The similar results for all age groups were obtained after analysis of MFI in the studied samples (Fig. 4b).

The expression of IFN-γ in NK cells of all age groups increased after their stimulation with IL-2 and PMA with ionomycin compared to the low level detectable in non-stimulated cells, i.e. 1.47 ± 0.13% in the young, 2.74 ± 0.7% in the old and 4.5 ± 0.95% in the oldest. In the young the expression of IFN-γ increased sixfold after stimulation with PMA and ionomycin, in the old 1.5-fold after stimulation with IL-2 and twelvefold after stimulation with PMA and ionomycin, in the oldest 1.33-fold after stimulation with IL-2 and nearly fivefold after stimulation with PMA and ionomycin (Fig. 4c). The results of MFI analysis were comparable to data presenting the percentages of cells with the expression of the studied protein in all age groups. In the young, however, a significant increase in the expression of IFN-γ was observed additionally after stimulation with IL-2 (Fig. 4d). Similarly to the other studied proteins, LPS did not affect the expression level of TNF or IFN-γ (Fig. 4a-d).

### Concentration of protein carbonyl groups and 8-isoprostanes in NK cell extracts from non-stimulated and stimulated NK cells of the seniors and the young

**Fig. 5:**
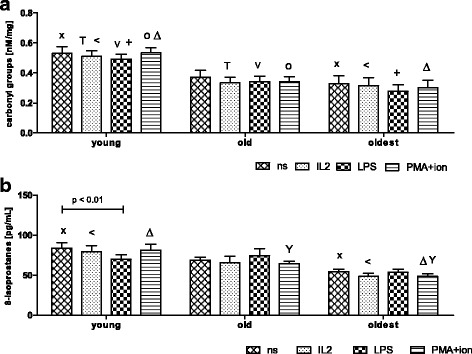
Concentration of protein carbonyl groups and 8-isoprostanes in cell extracts prepared from non-stimulated and stimulated NK cells of the young, the old aged under 85 and the oldest (aged over 85). Data are presented as means ± SEM. The same symbols over bars ^X^, ^<^, ^T^, ^o^, ^Δ^, ^V^, ^+^denote statistically significant differences between similarly treated NK cells of different age groups (i.e. young vs old; young vs oldest or old vs oldest). Horizontal lines above paired bars denote statistically significant differences between treated and untreated NK cells of the same age group (i.e. non-stimulated vs stimulated with IL-2, LPS or PMA with ionomycin). **a** The concentration of protein carbonyl groups in cell extracts of NK cells expressed as nmol/mg protein: ^Δ^, ^+^, ^o^*p* ≤ 0.01; ^x^, ^T^, ^<^, ^V^*p* < 0.05. **b.** The concentration of 8-isoprostanes in cell extracts of NK cells expressed as pg/mL: ^<^, ^Δ^, ^x^*p* ≤ 0.001; ^Y^*p* < 0.05

The highest concentration of carbonyl groups in the cultured NK cells was observed in the young (range 0.49 ± 0.03 – 0.53 ± 0.04). It was significantly higher compared to the old (range 0.33 ± 0.04 – 0.34 ± 0.03, except for non-stimulated cells) and the oldest (range 0.28 ± 0.04 – 0.33 ± 0.06). Analysis of the related samples, i.e. non-stimulated vs stimulated showed that the process of NK cell stimulation did not influence the level of oxidative stress in these cells, as there were no significant changes in the concentration of carbonyl groups after applied treatments in the studied age groups (Fig. 5a).

Similar results were obtained after the analysis of the concentrations of 8-isoprostanes in NK cell extracts. The highest concentration of 8-isoprostanes was observed in the young in most of the analyzed samples (range 70.00 ± 5.77 – 83.85 ± 6.86). It was significantly higher compared to the oldest (range 48.48 ± 3.86 – 54.36 ± 3.5), except for samples treated with LPS. However, there were no significant differences between concentration of 8-isoprostanes in NK cell extracts of the young and the old or old and the oldest, except for senior cells treated with combination of PMA and ionomycin (old vs oldest) (Fig. 5b). The analysis of the related samples, i.e. non-stimulated vs stimulated showed that process of NK cell stimulation did not influence the level of oxidative stress in these cells, as there were no significant changes in the concentration of 8-isoprostanes after applied treatments in the studied age groups. The only significant difference was observed between non-stimulated and LPS-stimulated NK cells of the young (Fig. 5b).

### Relationships between the analyzed parameters of NK cells stimulated in the same conditions within the studied age groups

Some relationships between expression levels of the analyzed cellular protective proteins, cytokines and concentrations of carbonyl groups and 8-isoprostanes in NK cells of the studied population were observed. Interestingly, very high positive correlations between SIRT1 and HSP70^intracellular^ in all NK cell samples, i.e. non-stimulated or treated with IL-2, LPS or PMA with ionomycin were noted. Low positive correlations were found also between the expression of SIRT1 and: (i) TNF (in all applied conditions), (ii) IFN-γ (in all conditions except for PMA + ionomycin), (iii) SOD2 (in samples stimulated with IL-2 and PMA with ionomycin) and HSP70^surface^ (in cells stimulated with IL-2) (Table 2).

**Table 2:** Correlation analysis of the study population

	Compared parameter (after stimulation in the same conditions)							
Parameter	Stimulation type	SOD2	SIRT1	HSP70^intr^	HSP70^surf^	TNF	IFN-γ	Carbonyl groups
SIRT1	none	ns						
	IL-2	0.222						
	LPS	ns						
	PMA/ion	0.231						
HSP70^intr^	none	ns	0.967					
	IL-2	0.235	0.957					
	LPS	ns	0.975					
	PMA/ion	0.315	0.926					
HSP70^surf^	none	0.694	ns	ns				
	IL-2	0.825	0.232	0.287				
	LPS	0.747	ns	ns				
	PMA/ion	0.652	ns	ns				
TNF	none	ns	0.406	0.445	ns			
	IL-2	ns	0.313	0.354	0.375			
	LPS	ns	0.390	0.403	0.276			
	PMA/ion	0.461	0.435	0.421	0.446			
IFN-γ	none	ns	0.415	0.464	ns	0.361		
	IL-2	ns	0.424	0.451	ns	0.301		
	LPS	0.257	0.371	0.421	0.369	0.368		
	PMA/ion	ns	ns	ns	ns	ns		
Carbonyl groups	none	ns	ns	−0.315	ns	ns	ns	
	IL-2	ns	ns	ns	ns	ns	ns	
	LPS	ns	ns	ns	ns	ns	− 0.346	
	PMA/ion	−0.313	ns	−0.33	ns	ns	ns	
8-Isoprostanes	none	ns	ns	−0.407	ns	−0.416	ns	ns
	IL-2	ns	ns	ns	ns	−0.437	ns	ns
	LPS	ns	−0.438	ns	ns	ns	ns	ns
	PMA/ion	ns	ns	ns	ns	ns	ns	ns

All values are presented as statistically significant (*p* < 0.05) Spearman’s correlation coefficients (R). ‘ns’ denotes statistically not significant. ‘*HSP70*^*intr’*^ intracellular HSP70, *‘HSP70*^*surf’*^ surface HSP70, *‘PMA/ion’* PMA + ionomycin

The expression of HSP70^intracellular^ revealed relationships similar to these of SIRT1. Low positive correlations were found between the expression of this protein and: (i) TNF (in all applied conditions), (ii) IFN-γ (in all conditions except for PMA + ionomycin), (iii) SOD2 in samples stimulated with IL-2 and PMA + ionomycin and (iv) HSP70^surface^ (in cells stimulated with IL-2) (Table 2).

HSP70^surface^ expression correlated with: (i) SOD2 (moderate to high correlation in all applied conditions), (ii) TNF (in all applied conditions except for non-stimulated NK cells) and (iii) IFN-γ (in cells stimulated with LPS). The expression of TNF correlated with: (i) SOD2 (in cells stimulated with PMA and ionomycin) and (ii) IFN-γ (in non-stimulated cells and cells treated with IL-2 and LPS). Low positive correlation was observed also between IFN-γ and SOD2 in cells stimulated with LPS (Table 2).

Some relationships were also observed between concentration of carbonyl groups or 8-isoprostanes and the other studied parameters. Carbonyl groups revealed weak, negative correlations with: (i) SOD2 (in cells stimulated with PMA and ionomycin), (ii) HSP70^intracellular^ (in non-stimulated and stimulated with PMA and ionomycin cells) and IFN-γ (in cells stimulated with LPS). Similarly, 8-isoprostanes showed weak, negative correlations with: (i) SIRT1 (in cells stimulated with LPS), (ii) HSP70^intracellular^ (in non-stimulated cells), TNF (in non-stimulated and stimulated with IL-2 cells) (Table 2).

### Relationships observed between the age and the analyzed parameters studied in non-stimulated and stimulated NK cells

Remarkably, the expression of SIRT1 and HSP70^intracellular^showed similar positive correlations with age in all studied variants, except for the NK cells stimulated with IL-2. The expression of TNF revealed rather low to moderate positive correlations with age in all applied experimental conditions. Interestingly, the levels of both carbonyl groups and 8-isoprostanes in NK cells correlated negatively with age in non-stimulated cells and in all variants of stimulation except for LPS in tests concerning 8-isoprostanes (Table 3).

**Table 3:** Correlation analysis of the study population: age vs the analyzed parameters

	Compared parameter								
	Stimulation type	SOD2	SIRT1	HSP70^intr^	HSP70^surf^	TNF	IFN-γ	Carbonyl groups	8-isoprostanes
Age	none	−0.225	0.301	0.300	ns	0.44	ns	−0.423	−0.584
	IL-2	ns	ns	ns	ns	0.478	0.242	−0.385	−0.685
	LPS	ns	0.417	0.409	ns	0.53	ns	−0.491	ns
	PMA/ion	ns	0.273	0.219	0.409	0.379	ns	−0.44	−0.555

All values are presented as statistically significant (*p* < 0.05) Spearman’s correlation coefficients (R). ‘ns’ denotes statistically not significant. ‘*HSP70*^*intr*’^ - intracellular HSP70; ‘*HSP70*^*surf*’^ - surface HSP70; ‘*PMA/ion*’ – PMA + ionomycin

## Discussion

The main finding of this study is that NK cells of the oldest seniors cultured for 2 days reveal high, constant level of stress response proteins expression, i.e. SIRT1 and HSP70 compared to seniors aged under 85 and the young people. Interestingly, the level of expression of SIRT1 and HSP70 in the oldest seniors did not change after stimulation of NK cells with various types of stimuli such as IL-2, LPS or PMA with ionomycin. On the contrary, the process of NK cell stimulation influenced significantly the expression level of SOD2 in the elderly, increasing with age of donors. The study shows also numerous correlations between the expression of cellular protective proteins in both stimulated and non-stimulated (control) NK cells and age of the study participants. Then, we provide some novel data concerning the influence of NK cell stimulation on the level of oxidative stress in these cells.

The study population was characterized by a slight increase in BMI observed in seniors, but the statistically significant differences were observed only between the young and both groups of seniors. The old and oldest participants did not differ significantly. These observations corresponded to the findings of the other authors who found that an increase in BMI was associated with ageing [47]. Then, the biochemical parameters of blood samples revealed some differences between age groups but they still remained within normal ranges [48]. These data support clinically reported good health of participants.

The results presented in the study correspond to our earlier observations concerning expression of cellular protective proteins in NK cells analyzed shortly after blood sample collection [46]. It is noteworthy that non-stimulated NK cells of the oldest seniors both briefly after isolation and after culturing for 48 h presented significantly higher expression levels of both SIRT1 and HSP70^intracellular^ compared to seniors under 85 and the young. Interestingly, in NK cells cultured for 48 h the expression of SIRT1 was even higher compared to freshly analyzed cells [46]. This might have been caused by the cell culture-induced state of oxidative stress [49, 50]. Our data concerning SIRT1 expression corresponded to the level of oxidative stress we found in cells cultured in vitro and freshly isolated ones [46].

On the contrary to SIRT1, the increased expression of HSP70^intracellular^ was higher in freshly isolated NK cells in contrast to the cultured ones [46]. This phenomenon may result from different characteristics of transcription patterns of both *SIRT1* and *HSP72* genes resulting from particular signaling pathways being under control of distinct transcription factors [30, 32, 33, 51]. Expression of chaperons that protect cells against cellular stress is under control of heat shock factor-1 (HSF1), which is activated within minutes after appearance of the stress factor, e.g. increase in temperature. The following increase in mRNA expression was observed within 6 h after triggering of cellular stress signaling pathway [32–34]. Kinetics of *SIRT1* gene expression is different as SIRT1 was detected within 24 h [52, 53] or 48-96 h after exposure to a stress factor [54, 55].

Interestingly, the expression of both SIRT1 and HSP70 in NK cells of the oldest did not change independently on the type of a stimulatory agent. On the contrary, in the young and elderly under 85, NK cells were sensitive to stimulation with IL-2 and PMA with ionomycin. These observations may suggest the role of hormesis in the process of ageing. Increasing levels of markers of oxidative stress and proinflammatory state are characteristics of the process of immunosenescence and can activate an adaptive stress response that may positively affect the lifespan [56]. Heat shock proteins and sirtuins are both involved in cellular adaptive response [29]. E.g. Wang et al. showed in endothelial progenitor cells that SIRT1 protein level increased in response to oxidative stress [53].

Intriguingly, the expression level of SOD2 in cultured, non-stimulated NK cells was quite low and comparable for all age groups. NK cells of the oldest seniors revealed, however, the highest sensitivity to stimulation compared to the other age groups. We observed higher expression of SOD2 in freshly isolated cells [46] and lower in cultured ones and this increase in SOD2 expression might have been caused by the change of cellular environment during process of NK cell isolation. This characteristic pattern of SOD2 expression can result from specific kinetics of *SOD2* gene transcription. The increase in mRNA synthesis is observed within 1-2 h after stimulation, the peak of synthesis occurs 4 - 6 h after stimulation and then SOD2 gene expression decreases [57, 58].

The pattern of surface HSP70 expression resembled to some extent that of SOD2. It was quite low in non-stimulated NK cells and differed only slightly between age groups presenting higher expression in seniors. NK cells of the oldest revealed the highest sensitivity to stimulation compared to the other age groups. According to Multhoff and colleagues, the commercially available antibodies are not able to distinguish integrated in the cell membrane HSP70 proteins but they rather detect HSP70 bound to TLR receptors on the cell surface [59]. Interestingly, in our studies lower and higher levels of HSP70^surface^ seemed to correspond to lower (in cultured) and higher (in freshly isolated) levels of HSP70^intracellular^ expression [46]. However, the accurate analysis of the origin of the extracellular HSP70 was out of scope of the presented study.

NK cells cultured for 48 h without stimulation showed very low level of TNF expression, comparable between the young and the old and slightly higher in the oldest. In all age groups NK cells were sensitive to stimulation with PMA and ionomycin. Increased production of proinflammatory cytokines in the process of ageing in mononuclear cells was observed earlier [60]. It was related to the proinflammatory state that accompanies the process of ageing, illustrated by the CRP values increasing with age [41]. This increase was also noted in the studied population but it did not exceed the normal range [46].

Similarly, cultured NK cells showed low, comparable levels of IFN-γ in the young and the old and higher expression in the oldest. This expression increased then significantly after activation; i.e. stimulation with PMA and ionomycin in all age groups. The weaker increase, but also significant in comparison to the young was observed after stimulation with IL-2 in the old and especially in the oldest seniors. These results are in line with observations described by Hayhoe et al. who showed significantly raised level of IFN-γ expression in NK cells of individuals aged more than 60 years compared to younger counterparts [61]. However, Krishnaraj reported diminished in the elderly secretion of IFN-γ after stimulation of NK cells with IL-2 for 18 h in comparison to the young but this deficiency appeared to be overcome by prolonged stimulation with IL-2 (7-day culture) [62]. Le Garff-Tavernier and colleagues noticed impaired production of IFN-γ in non-stimulated NK cells of the older subjects, but activated with IL-2 NK cells of the oldest seniors showed recovering of NK-cell function [43].

The process of ageing is accompanied by the increased levels of oxidative stress [63]. In this context, we obtained interesting results concerning the level of oxidative stress in NK cells which corresponded to our previous results regarding freshly isolated cells [46]. The highest concentrations of both carbonyl groups and 8-isoprostanes in NK cells were observed in the young and they differed significantly from the oldest. In spite of significant differences between the concentrations of carbonyl groups, their levels did not exceed normal ranges found in cell lysates, i.e. 1.3 nmol/mg in MRC-5-fibroblasts [64], and human plasma, i.e. 1.83 ± 0.4 nmol/mg [65], although they were definitely higher than in freshly isolated cells. Given the background from the meta-analysis of data concerning the measurement of 8-isoprostanes in different tissues and with the use of various methods, we found that results we obtained in both the young and the oldest were still within the normal range as depending on the tissue they might differ enormously. Erve and coworkers estimated that on average, the concentration of 8-isoprostanes detected in the plasma was 39.5 ± 36.2 pg/mL [66]. Thus, we could conclude that in spite of some differences between age groups in the level of markers of oxidative stress, i.e. carbonyl groups or 8-isoprostanes, they were still within the normal range. The most intriguing in this study was the lower level of markers of oxidative stress in the group of seniors, especially in the oldest, in the context of “Oxidative Stress Theory of Aging” [67]. However, Ristow and Zarse noted that ROS production within the mitochondria could cause an adaptive response and generate increased stress resistance which could result in a long-term reduction of oxidative stress. This type of retrograde response was named mitohormesis [68]. Then, Ristow and Schmeisser found that ROS can act as essential signaling molecules to promote metabolic health and longevity [69].

As we found no differences between the non-stimulated and stimulated samples (except NK cells treated with LPS in the young), we could draw the conclusion that different methods of NK cell activation did not influence the level of oxidative stress in the cells. When we compared our results from the present study with data obtained in freshly isolated cells, we also noted, that the levels of oxidative stress in NK cells cultivated 48 h in vitro were higher compared to the levels detected in freshly isolated cells [46]. These data might suggest that cell culture increased the level of oxidative stress in cultivated cells and this phenomenon was discussed by Professor Barry Halliwell [50]. However, the increased concentrations of carbonyl groups or isoprostanes remained within the normal range [64–66]. In this context, results concerning the elevated expression levels of cellular protective proteins, i.e. SIRT1 and HSP70, in the oldest, but not in the old or the young seemed to be even more interesting, because all the cells were cultured in the same conditions.

The present data related to the analysis of the relationships between the compared parameters corresponded to the results of our earlier study performed on freshly isolated NK cells [46]. Interestingly, positive correlations between two cellular protective proteins, i.e. SIRT1 and HSP70^intracellular^ were very high in cultured, both stimulated and non-stimulated NK cells. These relationships may corroborate the specific role of cellular protective proteins, involved in hormetic adaptive response [29]. Then, they correspond to results demonstrated by Westerheide et al., showing that SIRT1 directly deacetylates HSF1 (heat shock factor 1) and activates the cellular stress response [35]. The relationships between other protective proteins, i.e. SIRT1 and SOD2 showed rather low correlations in cells stimulated with IL-2 and PMA with ionomycin. Expression of SOD2 is under control of transcription factors of the FOXO family that promote the expression of stress response genes. SIRT1 activates several transcription factors of this family and some relationships between these two proteins have been reported [36, 56, 70].

We found rather low correlation coefficients between the expression of cellular protective proteins and cytokines in NK cells. The correlations were observed between SIRT1 and TNF (in all applied conditions) and between SIRT1 and IFN-γ (in all applied conditions except for PMA + ionomycin). NF-κB is a transcription factor responsible for the inflammatory response, which regulates expression of proinflammatory cytokines, e.g. IL-1, IL-6, TNF [15]. There is a regulatory loop between the expression of a cellular protective protein SIRT1 and the activity of transcription factor NF-κB, so that some relationships between SIRT1 and TNF have been reported [71, 72]. Of note, the synthesis of IFN-γ is under control of different transcription factors than TNF and subjected to the other mechanisms of regulation [73].

Interestingly, HSP70^surface^ revealed moderate to high positive correlation with SOD2. The expression of both proteins is controlled indirectly by SIRT1 so some positive feedback loops may also exist [35, 36, 56, 70]. Rather low positive correlations were observed also between HSP70^surface^ and TNF in all stimulated samples. These relationships may illustrate the proinflammatory state in activated NK cells characterized by increased level of TNF in response to increased level of surface HSP70.

Most of the analyzed parameters correlated with age, i.e. cellular protective proteins (SIRT1 and HSP70) and cytokines (TNF and IFN-γ) positively indicating their increase with age and markers of oxidative stress (carbonyl groups and 8-isoprostanes) negatively, showing their decrease with age. Our results correspond to data of the other groups concerning the increase in expression of SIRT1 in serum samples [74] and HSP70 in PBMCs [40] in the process of healthy ageing. They are also comparable with our previous results regarding the raise of concentration of proinflammatory cytokines; i.e. TNF and IL-6 in sera of the elderly [75]. The negative correlation between the level of oxidative stress and age seems to be surprising but it corresponds to conclusions presented by Ristow and Zarse concerning decrease in the level of oxidative stress resulting from cellular stress adaptive response to increased ROS formation in the process of ageing [68].

The advantage of our study is that although some relationships were already described earlier, i.e. between SIRT1 and HSP70, they concerned cell lines or animal models [35, 76]. We showed these relationships in cells isolated directly from human blood samples of participants recruited from different age groups. Moreover, we have demonstrated age-related changes in a specific population of lymphocytes, NK cells, which have yet not been studied regarding involvement of cellular protective proteins in the process of ageing.

## Conclusions

Our study provides new data concerning ageing of the human immune system and the role of NK cells in this process. We analyzed NK cells isolated from peripheral blood samples of different age groups. The cells were subjected to various types of stimulation to compare their influence on the expression of both cellular protective proteins involved in the adaptive stress response and cytokines, secreted by NK cells after activation. It was found that NK cells of the oldest seniors, aged over 85, presented constant, resistant to further stimulation, increased level of cellular protective proteins involved in stress response, i.e. SIRT1 and HSP70. We also observed very high positive correlations between SIRT1 and HSP70^intracellular^ in cultured, both stimulated and non-stimulated NK cells, suggestive of a specific role of these proteins in cellular adaptive response. On the contrary, SOD2 did not present the high, constant expression level in NK cells but revealed susceptibility to stimulation increasing with the age of participants. High positive correlations between SOD2 and HSP70^surface^ expression indicated a distinct role of SOD2 in cellular stress response in ageing process. Moreover, NK cells, of seniors did not show an increased level of oxidative stress markers such as isoprostanes and carbonyl groups.

In summary, our novel data show that NK cells of the oldest seniors reveal increased adaptation to stress response, a phenomenon that may contribute to the long lifespan of this group of the elderly.

## Abbreviations

ADL: Activities of daily living
CRP: C reactive protein
ERK: Extracellular signal-regulated kinase
FOXO: Forkhead transcription factor class O
GM-CSF: Granulocyte macrophage colony - stimulating factor
HSF: Heat shock factor
HSP: Heat shock protein
MCP: Macrophage chemoattractant protein
MFI: Mean fluorescence intensity
MIP: Macrophage inflammatory protein
MKK: Mitogen - activated protein kinase kinase
MMSE: Mini mental state examination
NF-κB: Nuclear factor kappa-light-chain-enhancer of activated B cells
NSAID: Non-steroid anti-inflammatory drug
PGC: Peroxisome proliferator - activated receptor gamma coactivator
PKC: Protein kinase C
PMA: Phorbol myristate acetate
RANTES: Regulated on activation, normal T cell expressed and secreted
SIRT: Sirtuin
SOD: Superoxide dismutase
STAT: Signal transducer and activator of transcription
TAC: Tetrameric antibody complexes
TLR: Toll-like receptor

## References

[1] Artis D, Spits H (2015). The biology of innate lymphoid cells. Nature.

[2] Cording S, Medvedovic J, Aychek T, Eberl G (2016). Innate lymphoid cells in defense, immunopathology and immunotherapy. Nat Immunol.

[3] Bryceson YT, Chiang SCC, Darmanin S, Fauriat C, Schlums H, Theorell J, Wood SM (2011). Molecular mechanisms of natural killer cell activation. J Innate Immun..

[4] Hazeldine J, Lord JM (2013). The impact of ageing on natural killer cell function and potential consequences for health in older adults. Ageing Res Rev.

[5] Sun JC, Ugolini S, Vivier E (2014). Immunological memory within the innate immune system. EMBO J.

[6] Vivier E, Raulet DH, Moretta A, Caligiuri MA, Zitvogel L, Lanier LL, Yokoyama WM, Ugolini S (2011). Innate or adaptive immunity? The example of natural killer cells. Science.

[7] De Maria A, Bozzano F, Cantoni C, Moretta L (2011). Revisiting human natural killer cell subset function revealed cytolytic CD56(dim)CD16+ NK cells as rapid producers of abundant IFN-gamma on activation. Proc Natl Acad Sci U S A.

[8] Fauriat C, Long EO, Ljunggren HG, Bryceson YT (2010). Regulation of human NK-cell cytokine and chemokine production by target cell recognition. Blood.

[9] Mariani E, Meneghetti A, Neri S, Ravaglia G, Forti P, Cattini L, Facchini A (2002). Chemokine production by natural killer cells from nonagenarians. Eur J Immunol.

[10] Yu TK, Caudell EG, Smid C, Grimm EA (2000). IL-2 activation of NK cells: involvement of MKK1/2/ERK but not p38 kinase pathway. J Immunol.

[11] Goodier MR, Londei M (2000). Lipopolysaccharide stimulates the proliferation of human CD56+CD3- NK cells: a regulatory role of monocytes and IL-10. J Immunol.

[12] Varma TK, Lin CY, Toliver-Kinsky TE, Sherwood ER (2002). Endotoxin-induced gamma interferon production: contributing cell types and key regulatory factors. Clin Diagn Lab Immunol.

[13] Mian MF, Lauzon NM, Andrews DW, Lichty BD, Ashkar AA (2010). FimH can directly activate human and murine natural killer cells via TLR4. Mol Ther.

[14] O’Connor GM, Hart OM, Gardiner CM (2006). Putting the natural killer cell in its place. Immunology.

[15] Bonizzi G, Karin M (2004). The two NF-κB activation pathways and their role in innate and adaptive immunity. Trends Immunol.

[16] Tamai R, Asai Y, Hashimoto M, Fukase K, Kusumoto S, Ishida H, Kiso M, Ogawa T (2003). Cell activation by monosaccharide lipid a analogues utilizing toll-like receptor 4. Immunology.

[17] Chopra RK, Nagel JE, Chrest FJ, Adler WH (1987). Impaired phorbol ester and calcium ionophore induced proliferation of T cells from old humans. Clin Exp Immunol.

[18] Good SR, Thieu VT, Mathur AN, Yu Q, Stritesky GL, Yeh N, O'Malley JT, Perumal NB, Kaplan MH (2009). Temporal induction pattern of STAT4 target genes defines potential for Th1 lineage-specific programming. J Immunol.

[19] Chang HC, Guarente L (2014). SIRT1 and other sirtuins in metabolism. Trends Endocrinol Metab.

[20] Elpek KG, Rubinstein MP, Bellemare-Pelletier A, Goldrath AW, Turley SJ (2010). Mature natural killer cells with phenotypic and functional alterations accumulate upon sustained stimulation with IL-15/IL-15Ralpha complexes. Proc Natl Acad Sci U S A.

[21] Kaszubowska L, Dettlaff-Pokora A, Hak L, Szarynska M, Ryba M, Mysliwska J, Mysliwski A (2008). Successful ageing of nonagenarians is related to the sensitivity of NK cells to activation. J Physiol Pharmacol.

[22] Liu Z, Kharmate G, Patterson E, Khan MM (2006). Role of H1 receptors in histamine-mediated up- regulation of STAT4 phosphorylation. Int Immunopharmacol.

[23] Wendt K, Wilk E, Buyny S, Buer J, Schmidt RE, Jacobs R (2006). Gene and protein characteristics reflect functional diversity of CD56dim and CD56bright NK cells. J Leukoc Biol.

[24] Bueno V, Sant’Anna OA, Lord JM (2014). Ageing and myeloid-derived suppressor cells: possible involvement in immunosenescence and age-related disease. Age (Dordr).

[25] Franceschi C, Bonafe M, Valensin S, Olivieri F, De Luca M, Ottaviani E, De Benedictis G (2000). Inflamm-aging. An evolutionary perspective on immunosenescence. Ann N Y Acad Sci.

[26] Barja G (2004). Free radicals and aging. Trends Neurosci.

[27] De la Fuente M, Miquel J (2009). An update of the oxidation-inflammation theory of aging: the involvement of the immune system in oxi-inflamm-aging. Curr Pharm Des.

[28] Calabrese EJ, Baldwin LA (2002). Defining hormesis. Hum Exp Toxicol.

[29] Calabrese V, Cornelius C, Dinkova-Kostova AT, Iavicoli I, Di Paola R, Koverech A, Cuzzocrea S, Rizzarelli E, Calabrese EJ (1822). Cellular stress responses, hormetic phytochemicals and vitagenes in aging and longevity. Biochim Biophys Acta.

[30] Hwang JW, Yao H, Caito S, Sundar IK, Rahman I (2013). Redox regulation of SIRT1 in inflammation and cellular senescence. Free Radic Biol Med.

[31] Saunders LR, Verdin E (2009). Stress response and aging. Science.

[32] Hensen SM, Heldens L, Van Genesen ST, Pruijn GJ, Lubsen NH (2013). A delayed antioxidant response in heat-stressed cells expressing a non-DNA binding HSF1 mutant. Cell Stress Chaperones.

[33] Östling P, Björk JK, Roos-Mattjus P, Mezger V, Sistonen L (2007). Heat shock factor 2 (HSF2) contributes to inducible expression of hsp genes through interplay with HSF1. J Biol Chem.

[34] Rossi A, Trotta E, Brandi R, Arisi I, Coccia M, Santoro MG (2010). AIRAP, a new human heat shock gene regulated by heat shock factor 1. J Biol Chem.

[35] Westerheide SD, Anckar J, Stevens SM, Sistonen L, Morimoto RI (2009). Stress-inducible regulation of heat shock factor 1 by the deacetylase SIRT1. Science.

[36] Hori YS, Kuno A, Hosoda R, Horio Y (2013). Regulation of FOXOs and p53 by SIRT1 modulators under oxidative stress. PLoS One.

[37] Morgan MJ, Liu Z (2011). Crosstalk of reactive oxygen species and NF-κB signaling. Cell Res.

[38] Owczarz M, Budzinska M, Domaszewska-Szostek A, Borkowska J, Polosak J, Gewartowska M, Slusarczyk P, Puzianowska-Kuznicka M (2017). miR-34a and miR-9 are overexpressed and SIRT genes are downregulated in peripheral blood mononuclear cells of aging humans. Exp Biol Med.

[39] Kovalenko EI, Boyko AA, Semenkov VF, Lutsenko GV, Grechikhina MV, Kanevskiy LM, Azhikina TL, Telford WG, Sapozhnikov AM (2014). ROS production, intracellular HSP70 levels and their relationship in human neutrophils: effects of age. Oncotarget.

[40] Njemini R, Bautmans I, Lambert M, Demanet C, Mets T (2007). Heat shock proteins and chemokine/cytokine secretion profile in ageing and inflammation. Mech Ageing Dev.

[41] Singh T, Newman AB (2011). Inflammatory markers in population studies of ageing. Ageing Res Rev.

[42] Gayoso I, Sanchez-Correa B, Campos C, Alonso C, Pera A, Casado JG, Morgado S, Tarazona R, Solana R (2011). Immunosenescence of human natural killer cells. J Innate Immun.

[43] Le Garff-Tavernier M, Beziat V, Decocq J, Siguret V, Gandjbakhch F, Pautas E, Debré P, Merle-Beral H, Vieillard V (2010). Human NK cells display major phenotypic and functional changes over the lifespan. Aging Cell.

[44] Folstein MF, Folstein SE, McHugh PR (1975). “Mini-mental state”. A practical method for grading the cognitive state of patients for the clinician. J Psychiatr Res.

[45] Katz S, Ford AB, Moskowitz RW, Jackson BA, Jaffe MW (1963). Studies of illness in the aged. The index of ADL: a standardized measure of biological and psychosocial function. JAMA.

[46] Kaszubowska L, Foerster J, Kaczor JJ, Schetz D, Ślebioda TJ, Kmieć Z (2017). Expression of cellular protective proteins SIRT1, HSP70 and SOD2 correlates with age and is significantly higher in NK cells of the oldest seniors. Immun Ageing.

[47] Hajek A, König HH (2017). The longitudinal association between informal caregiving and body mass index in the second half of life: findings of the German ageing survey. Public Health.

[48] Merck Manual Professional Version. Normal Laboratory Values. http://www.merckmanuals.com/en-pr/professional/appendixes/normal-laboratory-values/blood-tests-normal-values. Accessed 30 Jan 2018.

[49] Halliwell B (2007). Biochemistry of oxidative stress. Biochem Soc Trans.

[50] Halliwell B (2003). Oxidative stress in cell culture: an under-appreciated problem?. FEBS Lett.

[51] Chen X, Lu Y, Zhang Z, Wang J, Yang H, Liu G (2015). Intercellular interplay between Sirt1 signalling and cell metabolism in immune cell biology. Immunology.

[52] Kauppinen A, Suuronen T, Ojala J, Kaarniranta K, Salminen A (2013). Antagonistic crosstalk between NF- kB and SIRT1 in the regulation of inflammation and metabolic disorders. Cell Signal.

[53] Wang YQ, Cao Q, Wang F, Huang LY, Sang TT, Liu F, Chen SY (2015). SIRT1 protects against oxidative stress-induced endothelial progenitor cells apoptosis by inhibiting FOXO3a via FOXO3a ubiquitination and degradation. J Cell Physiol.

[54] Belloni L, Pollicino T, De Nicola F, Guerrieri F, Raffa G, Fanciulli M, Raimondo G, Levrero M (2009). Nuclear HBx binds the HBV minichromosome and modifies the epigenetic regulation of cccDNA function. Proc Natl Acad Sci U S A.

[55] Yun JM, Chien A, Jialal I, Devaraj S (2012). Resveratrol up-regulates SIRT1 and inhibits cellular oxidative stress in the diabetic milieu: mechanistic insights. J Nutr Biochem.

[56] Merksamer PI, Liu Y, He W, Hirschey MD, Chen D, Verdin E (2013). The sirtuins, oxidative stress and aging: an emerging link. Aging.

[57] Kamiński MM, Röth D, Sass S, Sauer SW, Krammer PH, Gülow K (1823). Manganese superoxide dismutase: a regulator of T cell activation-induced oxidative signaling and cell death. Biochim Biophys Acta.

[58] Kaszubowska L, Wierzbicki PM, Karsznia S, Damska M, Ślebioda TJ, Foerster J, Kmieć Z (2015). Optimal reference genes for qPCR in resting and activated human NK cells--flow cytometric data correspond to qPCR gene expression analysis. J Immunol Methods.

[59] Multhoff G, Hightower LE (2011). Distinguishing integral and receptor-bound heat shock protein 70 (Hsp70) on the cell surface by Hsp70-specific antibodies. Cell Stress Chaperones.

[60] Fagiolo U, Cossarizza A, Scala E, Fanales-Belasio E, Ortolani C, Cozzi E, Monti D, Franceschi C, Paganelli R (1993). Increased cytokine production in mononuclear cells of healthy elderly people. Eur J Immunol.

[61] Hayhoe RP, Henson SM, Akbar AN, Palmer DB (2010). Variation of human natural killer cell phenotypes with age: identification of a unique KLRG1-negative subset. Hum Immunol.

[62] Krishnaraj R (1997). Senescence and cytokines modulate the NK cell expression. Mech Ageing Dev.

[63] Cui H, Kong Y, Zhang H. Oxidative stress, mitochondrial dysfunction, and aging. J Signal Transduct. 2012. 10.1155/2012/646354. 10.1155/2012/646354 PMC3184498 21977319

[64] Sitte N, Merker K, Grune T (1998). Proteasome-dependent degradation of oxidized proteins in MRC-5 fibroblasts. FEBS Lett.

[65] Adams S, Green P, Claxton R, Simcox S, Williams MV, Walsh K, Leeuwenburgh C (2001). Reactive carbonyl formation by oxidative and non-oxidative pathways. Front Biosci.

[66] Van ‘t Erve TJ, Kadiiska MB, London SJ, Mason RP (2017). Classifying oxidative stress by F2-isoprostane levels across human diseases: a meta-analysis. Redox Biol.

[67] Hagen TM (2003). Oxidative stress, redox imbalance, and the aging process. Antioxid Redox Signal.

[68] Ristow M, Zarse K (2010). How increased oxidative stress promotes longevity and metabolic health: the concept of mitochondrial hormesis (mitohormesis). Exp Gerontol.

[69] Ristow M, Schmeisser S (2011). Extending life span by increasing oxidative stress. Free Radic Biol Med.

[70] Brunet A, Sweeney LB, Sturgill JF, Chua KF, Greer PL, Lin Y, Tran H, Ross SE, Mostoslavsky R, Cohen HY, Hu LS, Cheng HL, Jedrychowski MP, Gygi SP, Sinclair DA, Alt FW, Greenberg ME (2004). Stress-dependent regulation of FOXO transcription factors by the SIRT1 deacetylase. Science.

[71] Katto J, Engel N, Abbas W, Herbein G, Mahlknecht U (2013). Transcription factor NFκB regulates the expression of the histone deacetylase SIRT1. Clin Epigenetics.

[72] Yang H, Zhang W, Pan H, Feldser HG, Lainez E, Miller C, Leung S, Zhong Z, Zhao H, Sweitzer S, Considine T, Riera T, Suri V, White B, Ellis JL, Vlasuk GP, Loh C (2012). SIRT1 activators suppress inflammatory responses through promotion of p65 deacetylation and inhibition of NF-κB activity. PLoS One.

[73] Nakayama A, Kawasaki H, Jin C, Munekata E, Taira K, Yokoyama KK (2001). Transcriptional regulation of interferon gamma gene by p300 co-activator. Nucleic Acids Res Suppl..

[74] Kilic U, Gok O, Erenberk U, Dundaroz MR, Torun E, Kucukardali Y, Elibol-Can B, Uysal O, Dundar T (2015). A remarkable age-related increase in SIRT1 protein expression against oxidative stress in elderly: SIRT1 gene variants and longevity in human. PLoS One.

[75] Kaszubowska L, Kaczor JJ, Hak L, Dettlaff-Pokora A, Szarynska M, Kmiec Z (2011). Sensitivity of natural killer cells to activation in the process of ageing is related to the oxidative and inflammatory status of the elderly. J Physiol Pharmacol.

[76] Liu DJ, Hammer D, Komlos D, Chen KY, Firestein BL, Liu AY (2014). SIRT1 knockdown promotes neural differentiation and attenuates the heat shock response. J Cell Physiol.

